# Ocean-induced melt volume directly paces ice loss from Pine Island Glacier

**DOI:** 10.1126/sciadv.abi5738

**Published:** 2021-10-22

**Authors:** Ian Joughin, Daniel Shapero, Pierre Dutrieux, Ben Smith

**Affiliations:** 1 Polar Science Center, Applied Physics Lab, University of Washington, 1013 NE 40th Street, Seattle, WA 98105, USA.; 2 British Antarctic Survey, High Cross, Madingley Road, Cambridge CB3 0ET, UK.; 3 Lamont-Doherty Earth Observatory of Columbia University, 61 Route 9W, Palisades, NY 10964, USA.

## Abstract

The spatial distribution of ocean-induced melting beneath buttressing ice shelves is often cited as an important factor controlling Antarctica’s sea-level contribution. Using numerical simulations, we investigate the relative sensitivity of grounded-ice loss to the spatial distribution and overall volume of ice-shelf melt over two centuries. Contrary to earlier work, we find only minor sensitivity to melt distribution (<6%), with a linear dependence of ice loss on the total melt. Thus, less complex models that need not reproduce the detailed melt distribution may simplify the projection of future sea level. The linear sensitivity suggests a contribution of up to 5.1 cm from Pine Island Glacier over the next two centuries given anticipated levels of ocean warming, provided its ice shelf does not collapse because of other causes.

## Teaser

Glacier response to ice-shelf thinning is driven largely by the total melt rather than its spatial distribution.

## INTRODUCTION

**Fig. 1.**
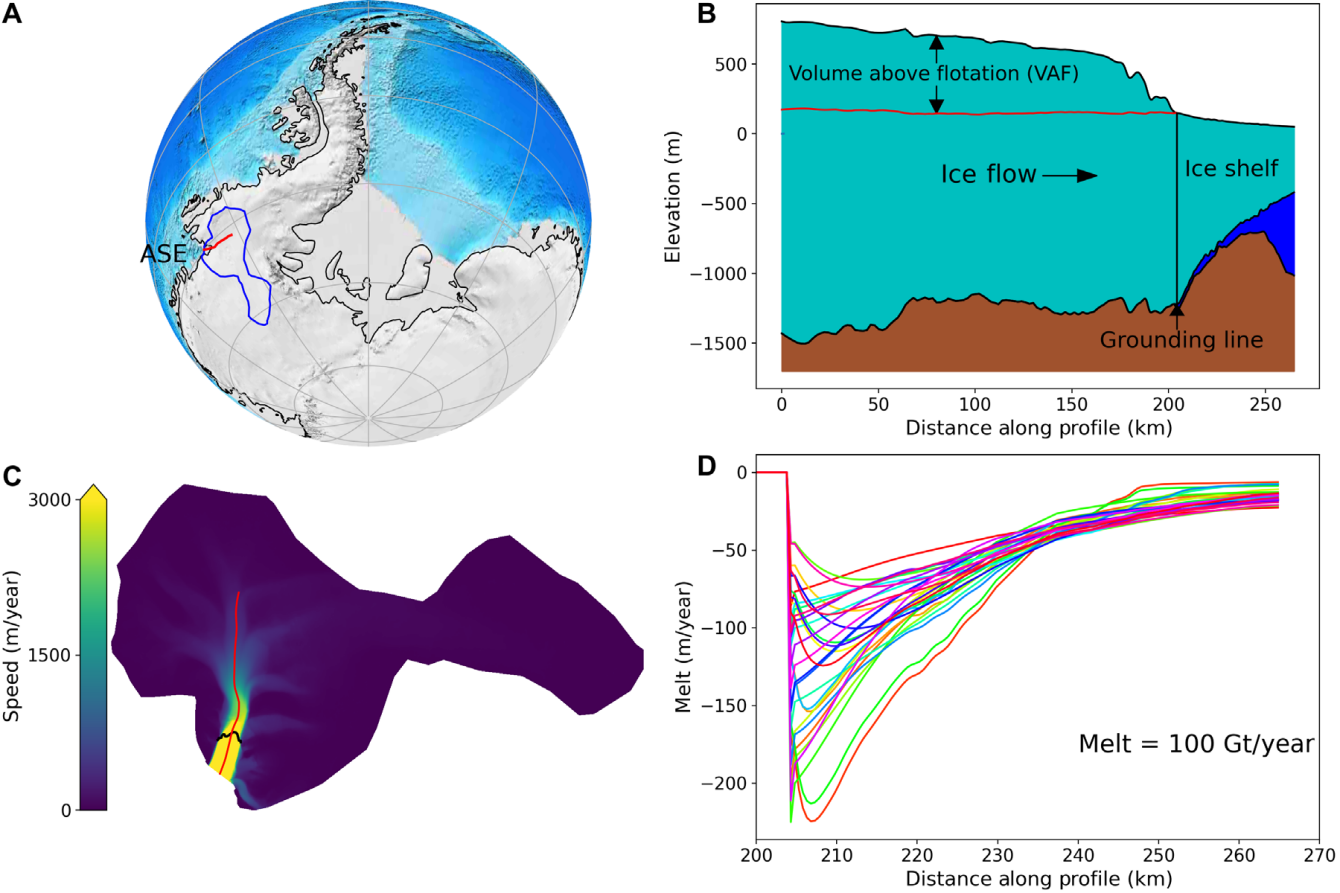
Pine Island Glacier location, flow line profile, model domain, and simulated melt profiles. (**A**) PIG model domain (blue outline) and the profile (red line) used in subsequent plots. (**B**) Initial profile geometry. Only the loss of ice volume above flotation (VAF) (red line) contributes to sea-level rise. (**C**) Observed 2017 speed used to initialize the model with the locations of the grounding line (black) and profile (red). (**D**) Initial melt-rate profiles for 30 realizations normalized to produce melt of 100 Gt/year.

Glaciers that discharge ice to Antarctica’s Amundsen Sea Embayment (ASE) (Fig. 1A) are losing mass at increasing rates (*1*), driven largely by enhanced oceanic melting beneath the floating ice shelves that restrain (buttress) their flow (*2*). This increased melting is caused by decadal- to centennial-scale trends in ASE winds that enhance on-continental-shelf transport of warm circumpolar deep water, increasing ocean heat content all the way to deep glacial grounding lines (grounded to floating ice transitions; Fig. 1B) (*3*, *4*). These melt-related processes cause considerable uncertainty in projections of the Antarctic contribution to sea level. In particular, these uncertainties are large for areas such as the ASE that are subject to marine ice-sheet instability, which occurs where ice rests on seafloor sloping downward toward the continent’s interior (*5*).

Although observations (*2*, *6*) and models (*7*–*9*) reveal that melt-induced ice-shelf thinning increases ice discharge, challenges remain in fully coupling ice and ocean models to resolve the spatiotemporal evolution of melt (*10*, *11*). Consequently, several studies have relied on depth-parameterized melt rates to drive ice-flow models (*7*, *8*, *10*, *12*, *13*), which, although often tuned to the initial state, may not hold as ice-shelf cavities evolve (*14*, *15*).

While several studies suggest that melt’s spatial distribution helps govern ice-sheet loss (*13*, *16*–*19*), it is not immediately clear that this should be so. Ice-shelf mass balance is determined by grounding-line flux and surface mass balance (SMB) (net snowfall after surface ablation) gains, which are countered by losses from subshelf melting and iceberg calving. Thus, when grounding-line flux increases, melting and calving must increase to compensate, or the shelf will thicken or lengthen, slowing flow. Once an ice column crosses the grounding line, it essentially integrates the melt distribution as it flows seaward, so the spatial pattern may be less important than the integrated melt. We evaluate this hypothesis using numerical simulations of rapidly thinning [0.13 to 0.17 mm/year sea-level equivalent (sle)] Pine Island Glacier (PIG; Fig. 1) (*20*, *21*).

## RESULTS

We simulated PIG with a shallow-shelf, ice-flow model initialized with velocity from 2017 (Fig. 1C and Materials and Methods). Unless noted otherwise, the simulations used regularized Coulomb friction (RCF) for glacier sliding, which best reproduces PIG’s recent behavior (*22*). To evaluate sensitivity to melt distribution, each simulation used a prescribed shelf-integrated melt total, with a randomly generated, depth-parameterized spatial distribution (e.g., Fig. 1D and Materials and Methods). Although quantitatively these distributions vary greatly, qualitatively they maintain observed characteristics (e.g., a tendency for greater melting at depth).

**Fig. 2.**
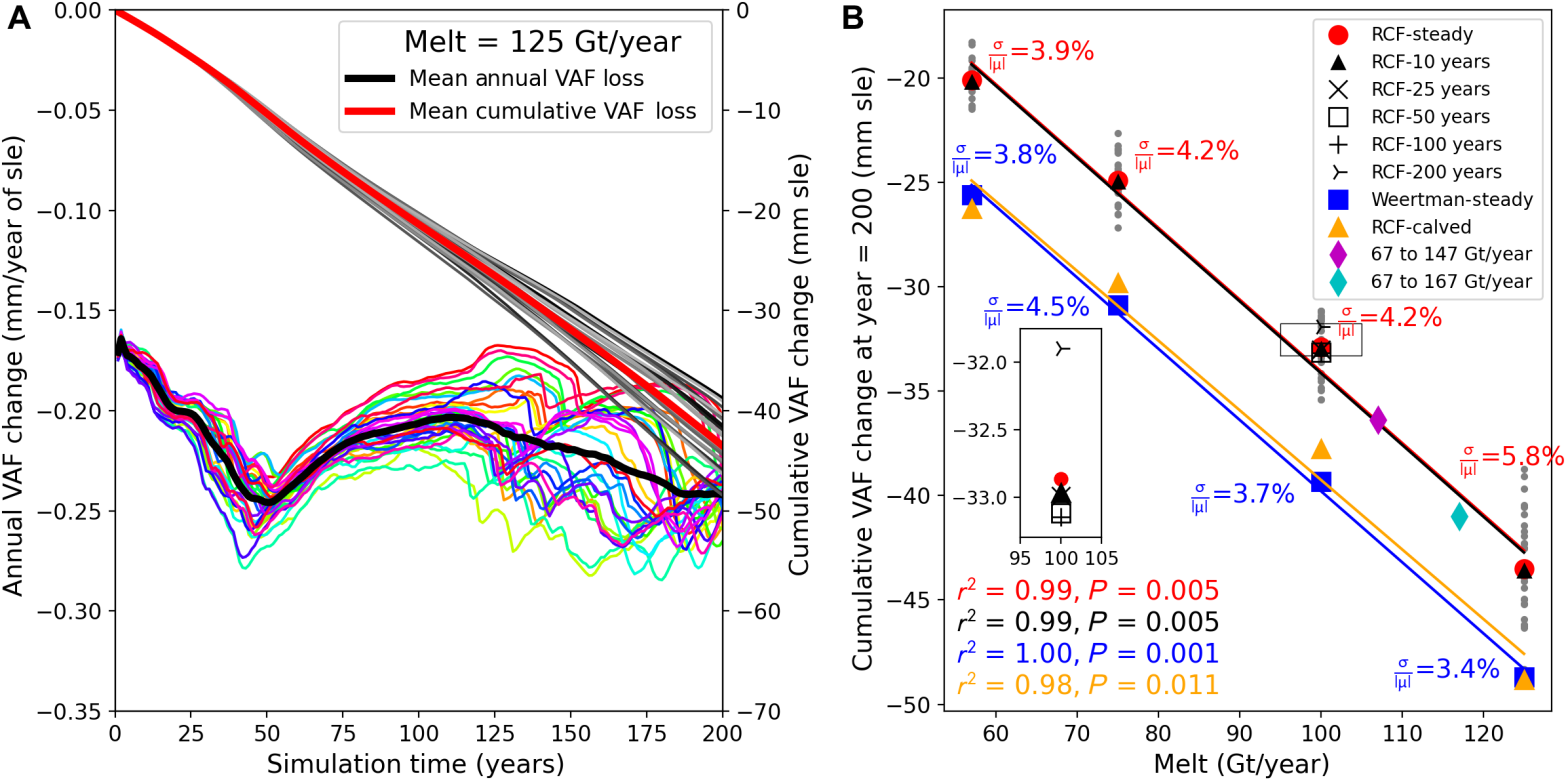
Results from simulations of PIG with prescribed melt values. (**A**) Simulated annual (colors) and cumulative individual (grays) changes in sea-level equivalent (sle) units for thirty 200-year simulations with 125 Gt/year steady melt. (**B**) Mean loss as a function of melt for five cases: steady melt, decadal modulation, variable periodicity (100 Gt/year only), Weertman sliding, and RCF calved (2020 shelf extent). Gray dots show individual RCF-steady realizations. Normalized SDs $\left(\frac{\sigma }{|\mu |}\right)$ for the steady cases with the RCF and Weertman parameterizations are also shown. Inset shows detail for the box centered on 100 Gt/year. The 30-realization means are well represented (*r*^2^ = 0.98 to 1.00) by linear fits (lines). Results from simulations, where melt increased linearly from 67 to 147 Gt/year (magenta) and 67 to 167 Gt/year (cyan), are also shown.

We simulated PIG evolution for 200 years, using four melt-rate values [57, 75, 100, and 125 Giga tons (Gt)/year] each applied to 30 randomly generated melt distributions (e.g., Fig. 1D). Figure 2A shows the annual and cumulative volume above flotation (VAF; Fig. 1B) losses for the 125 Gt/year case, which reveals only minor distribution-related variations as the grounding line retreated over bed protrusions.

To examine temporal melt variability, we simulated behavior with the same 200-year melt averages, but with a ±50% sinusoidal modulation (10-year period) to emulate observed decadal-scale variation (*4*). The decadal-scale forcing results are virtually identical to the steady-melt cases, as is the case for longer periods (25, 50, 100, and 200 years; Fig. 2B, inset).

To investigate sensitivity to the basal friction model, we repeated the steady-melt experiments with Weertman instead of RCF sliding (*23*). This change produced an additional sea-level rise of ~5 mm after 200 years.

Across all simulations, the sensitivity of ice loss to individual distributions is minor (2.8 to 5.8%), as indicated by the ratios of the 30-realization SDs (σ) to means (μ; see Fig. 2B for steady-melt ratios). When the integrated melt is varied for the different sets of simulations, the ensemble-mean cumulative losses (Fig. 2B) increase linearly (*r*^2^ ≥ 0.98), indicating that melt paces ice loss. Linear fits to the ensemble members (e.g., gray points in Fig. 2B) rather than ensemble means yield the same slopes, but the variability within the ensemble reduces the *r*^2^ values slightly (0.94 to 0.97).

**Fig. 3.**
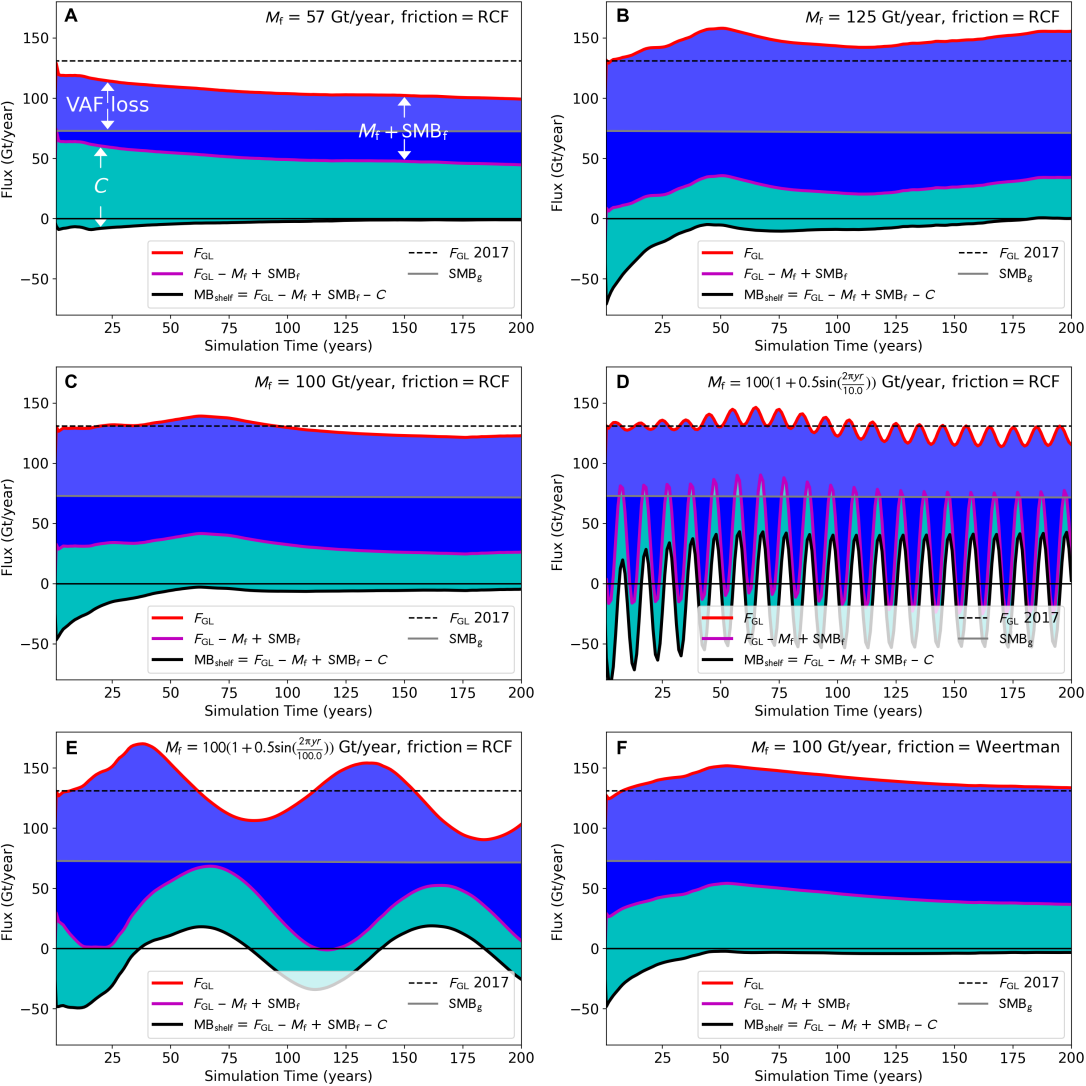
Ice-shelf mass balance (MB_shelf_; black line) and its constituent terms: grounding-line flux (*F*_GL_), melt and SMB (*M*_f_*+* SMB_f_; dual blue shading), and calving (*C*; cyan region). (**A**) Steady *M*_f_ *=* 57 Gt/year and (**B**) steady *M*_f_ *=* 125 Gt/year simulations. Cases with *M*_f_ *=* 100 Gt/year for (**C**) steady, (**D**) 10-year, and (**E**) 100-year periods and (**F**) Weertman sliding. The dashed black line indicates *F*_GL_ observed in 2017. The VAF loss is the difference between *F*_GL_ (red) and grounded SMB (SMB_g_; gray) curves. All curves are 30-realization averages.

As hypothesized above and confirmed by our simulations, a buttressed glacier only sustains increased discharge if its ice shelf sheds the excess mass through melting and calving. To further demonstrate, Fig. 3 shows simulated ice-shelf mass balance. Ice-shelf thinning is initially around −10 Gt/year (black curve Fig. 3A) for melting of 57 Gt/year but declines to nearly zero after 100 years. By contrast, with 125 Gt/year melt (Fig. 3B), the shelf thins substantially for several decades, which results in a final VAF loss rates about a third above present. The 100 Gt/year simulation (Fig. 3C) produces intermediate results with ice loss similar to present throughout the simulation. For the periodic simulations, the shelf oscillates between thickening and thinning, with VAF losses lagging the melt forcing by less than a decade. Weertman sliding increases ice discharge, which is compensated for by a greater calving rate (compare cyan regions in Fig. 3, C and F).

In all steady-melt cases, ice-shelf thinning declines over the course of the simulation to relatively low rates (<~10 Gt/year), representing a small fraction of the melt. Although small, these losses are critical for sustaining grounding-line retreat by consuming newly ungrounded ice.

**Fig. 4.**
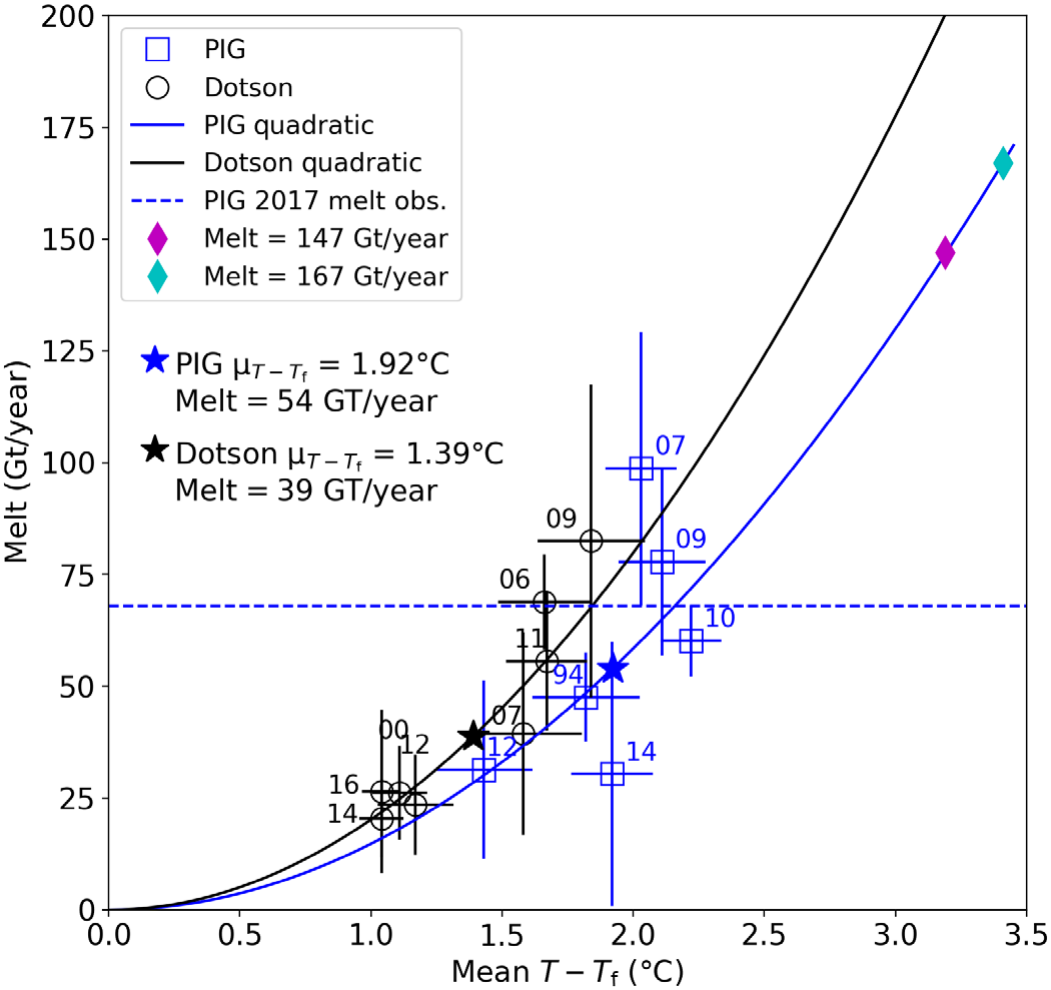
Melt rates for PIG and Dotson ice shelves estimated from summer ocean hydrographic sections (*4*). Solid lines show quadratic relation for melt, and stars indicate the mean observed temperature. The dashed line shows a 2017 PIG melt estimate from remote sensing (Materials and Methods). Magenta and cyan diamonds correspond to their Fig. 2 counterparts after 200 years.

Figure 4 shows estimated melt at several times since the 1990s for the PIG (blue) and nearby Dotson (black) ice shelves, using summer ocean hydrographic sections. These data indicate a quadratic relation between melt and temperature above freezing (*4*, *24*). We estimated a mean melt nominally for 2017 using remote sensing data, which we take as a proxy for the average melt over the last decade (see Materials and Methods). This value of 67 Gt/year corresponds to 2.15°C above freezing (slightly above the observed mean in Fig. 4), and it falls between our 57 and 75 Gt/year simulations.

The simulations indicate that losses should decline by 2020 for melt in the range of 57 to 75 Gt/year, with 100 Gt/year of melt needed to sustain 2017 losses (Fig. 3C) over the following decade. In actuality, however, ice loss has increased by ~10% since 2017 as the glacier sped up, coincident with a ~20-km shelf-length reduction due to iceberg calving (*25*).

To investigate its effect, we simulated the recent shelf retreat as an instantaneous calving event by regridding the 2017 initial conditions to match the 2020 geometry (Materials and Methods and fig. S4). With this shelf reduction included, the 57 and 75 Gt/year cases produce speeds consistent with 2020 observations. When propagated forward 200 years, this model projects an additional ~5 mm sle loss for all melt levels (Fig. 2B).

## DISCUSSION

Contrary to earlier results using idealized one-dimensional (1D) models (*16*, *17*), our simulations indicate that the rate of ice loss is relatively insensitive (<~6%) to melt distribution, which may be due to several factors. First, both earlier works used spatially dependent melt distributions rather than depth-dependent distributions. As a consequence, ice shelves in those models cannot respond by thinning to alter the melt intensity near the grounding line. Thus, high melt at a point near the grounding line can only be reduced by grounding-line retreat. By contrast, in our model and most other models, the simulated ice shelf can thin to redistribute melt without necessarily causing the grounding line to retreat. In addition, one of the earlier studies fixed the melt rate while allowing the total melt to increase as the shelf extent increased (*16*), so the total melt may have been an important factor for that model as in our results. The other of the two studies fixed the total melt by altering the shelf length (*17*), which introduced an additional calving term that reduces the ice-shelf volume to a similar extent that more melt would have (Fig. 3). Last, both 1D models are fed by a fixed inflow of ice from a relatively short distance upstream of the grounding line. For PIG, however, a broad interior basin converges on the narrow main trunk, allowing inflow to the trunk to increase as the glacier speeds up. Unlike the case for models with fixed inflow, this extra inflow can moderate rates of grounding-line thinning and retreat (*7*).

The simulations indicate that cyclic melt variability at decadal through centurial time scales has little effect (Fig. 2B), suggesting that observed natural decadal-scale melt variability matters little for century-scale projections (*3*, *4*), with only the long-term average melt rate driving the sea-level rise. To the extent that oceanic variability might influence melt behavior (*26*), it would appear to do so through its influence on mean melt rates. Despite the model’s complexity, a single value, the 200-year melt-rate average, almost entirely predicts PIG’s 200-year contribution to sea level (0.34 mm per Gt/year of average melt). Last, neither Weertman sliding nor a reduced shelf extent alters this sensitivity, but each adds ~5 mm to the 200-year increase in sea level. We note that while solid-Earth and sea-level feedbacks can influence ice loss, they should have only a negligible effect at the loss rates and time scales that we examine here (*27*), so our model does not include these processes.

Although our experiments were designed as sensitivity experiments, they can help assess PIG’s 200-year melt-related sea-level contribution. First, we consider how melt might evolve over this period. In the decades before 2009, PIG’s grounding line retreated from a bathymetric high (Fig. 1B) to its trough’s deeper reaches (*28*), where water has higher melt potential, so that not all recent melt increases are due to externally forced increases in ocean heat content (*14*, *29*). For the next two centuries, however, melt will likely be driven by ocean heat content because the grounding line should remain at a similar depth as it retreats over smaller bed elevation variations (Fig. 1B).

At decadal time scales, ocean heat content variability appears associated with changes in shelf-break wind anomalies emanating from the tropics (*24*). At multidecadal time scales, anthropogenically forced wind changes have produced a potential melt trend of 20 Gt/year per century for PIG, which climate models suggest should continue through the 21st century (*3*). Doubling this trend to achieve an approximate bound yields a 200-year increase from 67 to 147 Gt/year (107 Gt/year average), equivalent to ocean warming of 1.04°C (Fig. 4). Simulations with this trend yield 36 mm sle (Fig. 2B). Alternatively, a 33-model average from Coupled Model Intercomparison Project 5 (CMIP5) indicates a 21st century warming of 0.63°C at 400 to 800 m depth on the ASE continental shelf (*10*), equivalent to a 50 Gt/year per century trend. Extrapolating this trend to 200 years is equivalent to warming of 1.26°C (Fig. 4; 117 Gt/year average), which produces 41 mm sle when simulated (Fig. 2B). Adding potential contributions from the 2020 shelf extent and Weertman friction, the maximum loss would be ~51 mm sle.

The linearity evident in Fig. 2 indicates that these estimates can be easily revised up or down as better estimates of warming in the ASE become available. An important assumption here is that PIG’s ice shelf remains largely intact. A complete surface melt–induced collapse would yield greater losses (*30*), but such an event is unlikely to occur because of surface warming at least through the 21st century (*31*). The recent shelf loss appears to be due to mechanical weakening in response to the speedup, and the likelihood that such processes will drive further retreat is unclear (*25*, *32*).

**Fig. 5.**
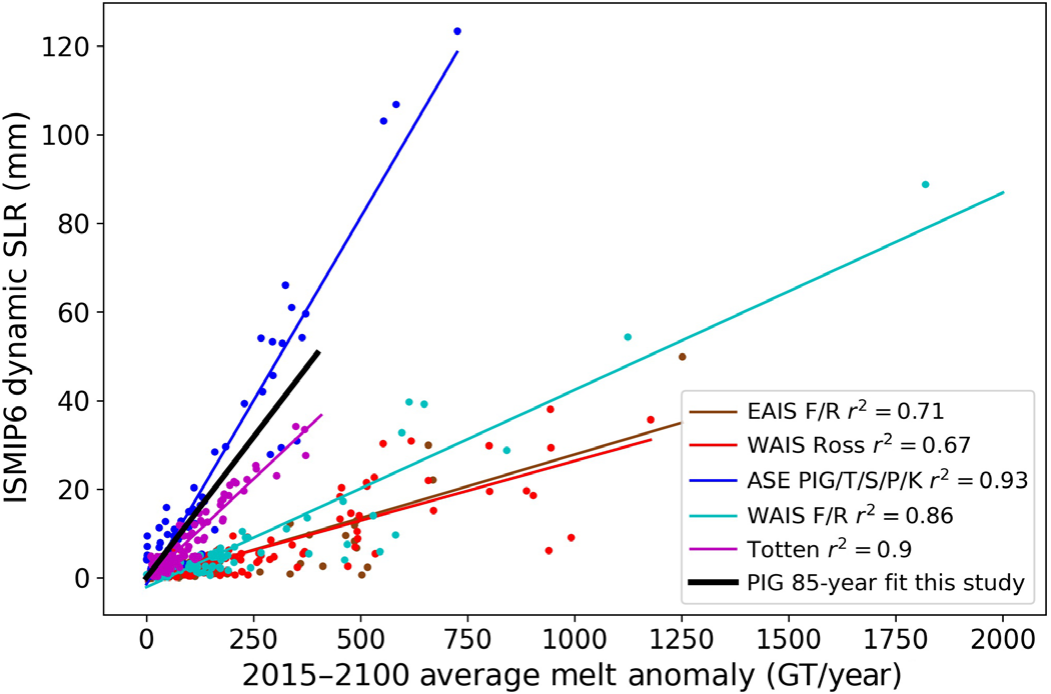
Additional ice loss (color) for the 85-year ISMIP6 model runs relative to control runs in response to melt forcing in excess of that used in each model’s respective control run (*13*). We computed the 85-year response from our model (black) and treated the 57 Gt/year result as the control run. For this comparison, the fit to our result is extrapolated over a much greater range of melt values than we evaluated. Note that the area referred to as “Totten” glacier in this figure also includes the Moscow, Frost, and several smaller glacier catchments (*13*). SLR, sea level rise; WAIS, West Antarctic Ice Sheet; ASE, Amundsen Sea Embayment; PIG, Pine Island Glacier; T/S/P/K, Thwaites/Smith/Pope/Kohler; WAIS F/R, WAIS sector of the Filchner-Ronne Ice Shelf; EAIS, East Antarctic Ice Sheet.

Our results indicate that centennial-scale increases in sea level depend directly on the spatiotemporal melt average, with little sensitivity to spatial or temporal variability about this mean. While our hypothesis was verified for one glacier, it likely holds for other glaciers with well-confined shelves. To help validate this supposition, Fig. 5 shows ice loss as a function of melt for the sectors of Antarctica that the Ice Sheet Model Intercomparison Project for CMIP6 (ISMIP6) indicates will produce the most loss over the 21st century (*13*). Similar to our results, these multimodel ensembles indicate that ice loss responds linearly to total melt for the ASE and other ice-sheet sectors that are subject to large melt-forced losses (Materials and Methods) (*13*). The *r*^2^ values for the ISMIP6 data are not as high as for our single-model results, which is not surprising given the variance likely introduced by the variety of models and forcings used to generate the results. The relatively high *r*^2^ values, however, suggest that the spread in the projections from these models is largely driven by differences in the way they each simulate melt, rather than by their respective implementations of the ice dynamics (*13*). Our results are also consistent with other studies that find a nearly linear response of ice loss for a doubling or halving of the melt rate using the ISMIP6 models (*33*, *34*). Thus, the linear relation between average melt and loss at century scales in both our model and the ISMIP6 models supports a linear response approach to projecting sea-level rise (*33*).

Over the next century, the West Antarctic Ice Sheet will likely produce the bulk of Antarctica’s contribution to sea-level rise (*13*), with PIG currently accounting for ~40% of West Antarctic losses (*20*). Our maximum estimate of 51 mm sle for the next two centuries with the current shelf configuration translates to less than 20 mm sle for the 21st century, which is well within the range of current consensus estimates (*35*). To the extent that other models produce proportionately larger ocean-forced losses from the ASE, they do so by simulating far greater melt rates (e.g., Fig. 5), which analysis of recent trends does not support (*3*). While our estimate represents a moderate contribution to sea level, PIG’s main trunk still thins by several hundred meters over 200 years. Furthermore, a quadratic dependence of melt on temperature indicates that PIG and other glaciers in the ASE are primed for large losses beyond 200 years, which could lead to a complete collapse of the West Antarctic Ice Sheet over periods as short as several centuries.

## MATERIALS AND METHODS

### Model software

As described below, we numerically solve the diagnostic, depth-averaged, shallow-shelf equations for ice-flow velocity (*36*) and the prognostic equations of continuity for ice thickness, using the open-source ice-sheet modeling package, icepack (*37*, *38*), which is built using the finite element package firedrake (*39*). The forward and inverse models based on icepack are available online (*40*) along with supporting routines (*41*).

The key feature of icepack that is relevant to this work is its flexibility with respect to changing model parameterizations (*38*). Individual components of the model physics can be substituted or altered. In this work, we used alternative friction laws. Icepack also includes a state-of-the-art inverse problem solver based on the Gauss-Newton algorithm. This solver converges faster and with greater reliability than the more commonly applied gradient descent and Broyden-Fletcher-Goldfarb-Shanno methods. The physics models and numerical solvers in icepack are tested by comparing numerical and analytical solutions on standard test cases and checking for the expected order of convergence of the numerical solutions as the grid is refined (*38*).

### Friction law

An RCF function was used in an earlier study (*22*) to relate sliding speed, *u*, to basal shear, τ_b_, that is given by

$$\tau_{b}=-C{\left(\frac{|u|}{|u|+u_{0}}\right)}^{1/m}\frac{u}{|u|}$$ (1)

where *m* = 3. In fixing *u*_0_, the model approximates Coulomb friction over the fast-moving (*u* > *u*_0_) parts of the model domain, while producing more traditional Weertman-like behavior for the slower moving areas.

Instead of the RCF function given in Eq. 1, we used the following related function

$$\tau_{b}=-C{\left({|u|}^{1+1/m}+u_{0}^{1+1/m}\right)}^{-1/(m+1)}{|u|}^{1/m-1}u$$ (2)

This function has an antiderivative that is much easier to express than the antiderivative of the RCF function, which involves hypergeometric functions. When the friction function has an easily expressed antiderivative, it is possible to apply much more efficient and robust numerical solvers. Figure S1 shows both functions with a value of *u*_0_ = 300 m/year, where *C* has been chosen so that both functions produce the same arbitrary value of τ_b_ at the upper end of the speed range. Qualitatively, the character of both curves is similar, but with some differences in the middle of the range of speeds. For the most part, these differences can be eliminated by adjusting the value of *u*_0_. As fig. S1 indicates, a value of *u*_0_ = 300 in Eq. 2 produces nearly identical behavior as with *u*_0_ = 200 m/year in Eq. 1. We used a value of *u*_0_ = 300 in Eq. 2 for all of the RCF simulations.

Over most of the model domain, the value of *C* stays constant with time throughout the simulation. An exception occurs for the area near the grounding line, where *C* decreases linearly as a function of changes in the evolving height above flotation such that it tends toward zero as the ice approaches flotation (*7*). We picked the threshold height, *h*_T_, which best matches recent observations using the same procedure as in an earlier study (*4*). Despite the slight differences in the friction laws used for the two studies (fig. S1), we obtained similar values of *h*_T_ (41 and 46 m for the current and prior studies, respectively).

We also implemented a Weertman sliding law of the form

$$\tau_{b}=-C{|u|}^{1/m}\frac{u}{|u|}$$ (3)

As with the RCF case, we use the threshold, *h*_T_, that best reproduces recent behavior. Although the earlier study used a different implementation of the shallow-shelf equations (*22*), the thresholds were nearly identical (122 m for this study versus 123 m for the other).

### Inverse model

We initialized the model to 2017 conditions by solving for the friction-law coefficient (*C* in Eqs. 1 and 3) on the initially grounded ice, while assuming that its value is zero beneath the floating ice. For the grounded ice, we used the temperature-dependent value of the Glen’s flow law coefficient (*3*), *A*, which was used in an earlier study (*7*). On the floating ice, we inverted directly for *A*, which implicitly includes the effects of damage enhancement. For a narrow region (a few kilometers) above the grounding line, we solved for both *A* and *C* to smooth out any discontinuities between the forward and inverse solutions for *A*. As is common practice, the model actually solves for *C* = β^2^ and *A* = *e*^θ^.

The solutions for *A* and *C* are iterative and interleaved at each step. Specifically, the first step in the solution is for *C* and that value is used to obtain a new solution for *A* and so forth until the model converges (*7*). The model uses Tikhonov regularization for determining both *A* and *C*. For the solution for *C*, we used an L-curve approach to pick the regularization coefficient (fig. S2). We used a similar approach for *A* but selected a little more regularization than we might have otherwise to avoid unrealistically enhanced values of *A* in the shear margins, which increased the residual misfit by ~4 m/year (22 to 26 m/year).

### Forward model

We initialized the forward model with the solutions for *A* and *C* from the inversion. At each time step, the diagnostic equations are solved to determine ice-flow velocity, followed by a prognostic solve for thickness. After thickness is determined, regions that are afloat are determined by evaluating where surface elevation is at or below the height above flotation.

A time step of 0.05 years was used for the lower melt (57 and 75 Gt/year) simulations, and a value of 0.025 years was used for the high-melt, faster-flow cases (100 and 125 Gt/year). We used a fully implicit Lax-Wendroff time-stepping scheme, which guarantees second-order convergence in time (*42*). Using an implicit-in-time method also ensures stability for any time step. Nonetheless, we used a time step that is close to that prescribed by the Courant-Friedrichs-Lewy condition to maintain acceptably low errors.

### Model domain

The finite element mesh (fig. S3) consists of 107,480 triangular elements, with 54,123 vertices (*43*). Mesh resolution is set to approximately 300 m for the part of the main trunk over which grounding-line retreat occurs. We developed the simulations using piecewise linear basis functions, which yield much quicker solutions. The final solutions were all run with more accurate piecewise quadratic basis functions.

### Velocity observations

We constrained the inversions described above using a map representative of average flow for 2017 (Fig. 1C). For the rapidly evolving, fast-moving regions, we averaged all of the available Sentinel-1A/B data for 2017. This coverage did not include some of the slow-moving regions of the upper catchment. For these areas, we substituted speeds from MEaSUREs Version 2 velocity map of Antarctica (*44*, *45*) to create a merged map (*43*).

### Observed surface and bed topography

We initialized the model with the same elevation time series used in an earlier study (*22*), which is based on Digital Elevation Models created from WorldView stereopairs and numerous airborne lidar surveys from NASA’s Operation IceBridge. We also used an updated time series for estimating melt rates, which excludes data after calving in late 2017 to avoid introducing thinning artifacts near the shelf front (*43*).

We used the BedMachine Antarctica Version 2 dataset to determine the bed geometry in the model (*46*, *47*). We made minor adjustments near the grounding line to ensure that the grounding-line thickness data used in the model were self-consistent (*43*). Specifically, using the 2017 surface elevation, we adjusted the bed model to force the grounding line to its nominal 2017 position by lowering the bed geometry, where it inconsistently indicated grounded ice (*22*). We did not have a precise measurement of the grounding line for 2017, so we derived an approximate position using TerraSAR-X data (*48*).

### Observed SMB

We used an estimate of SMB (net snowfall accumulation) derived from airborne radar observations with constraints from ice cores (*21*, *43*), which is held fixed throughout the simulations.

### Melt estimate for 2017

The rate of change in thickness, *H*, for an ice shelf with flow speed, **u**, is given by

$$\frac{\partial H}{\partial t}=-\nabla ∙(Hu)+\text{SMB}-M_{b}$$ (4)

where the three terms on the right-hand side represent flux divergence, SMB, and basal melt, respectively. In this calculation, the ice column is assumed to be solid ice (i.e., it has been corrected for firn-air content as is the case in the simulations). Using the 2017 velocity and thickness datasets, the flux divergence is 55 Gt/year. Combined with the SMB for the shelf (2 Gt/year), the steady-state $\left(\frac{\partial H}{\partial t}=0\right)$ melt rate for the shelf is 57 Gt/year. Here, steady state refers to the conditions necessary to maintain the ice-shelf thickness based on the inflow from the grounded regions, which presently are far from a steady state.

For the period from 2009 to 2017 when speeds were relatively steady (*25*), we computed a mean annual thinning of 10 Gt/year using yearly estimates of thickness derived from surface elevation under the assumption of hydrostatic equilibrium. The SD of this estimate is 9 Gt/year, which represents both noise and interannual variability. Given this variability, we used the 8-year thinning average to compute a melt rate of 67 Gt/year, which we take to be representative of 2017 conditions. Our 57 and 75 Gt/year simulations provide approximate 1-σ bounds about this mean.

### Melt realizations

We generated 30 independent realizations for each nominal melt value (*43*). For each realization, the melt is represented by a thickness (depth)–dependent three-section piecewise linear function beneath the floating ice as

$$m(H)=\text{smooth}(\{\begin{array}{cc} \alpha_{1}H+\beta_{1} & H\leq H_{1}\\ \alpha_{2}H+\beta_{2} & H_{1}<H\leq H_{2}\\ \alpha_{3}H+\beta_{3} & H_{2}\leq H \end{array},\gamma ,\text{mode})\;\text{and}$$

$$M(t)=\xi \int_{\text{shelf}}m(H)\mathrm{dS}$$ (5)

A uniform distribution was used to generate all randomly selected parameters. The ranges for *H*_1_ and *H*_2_ were (300 to 625) and (650 to 1050) m, respectively. The ranges for α_1_, α_2_, and α_3_ were (−0.1 to −0.3), (−4 to −0.5), and (−4 to −0.5) year^−1^, respectively. The intercept, β_1_, randomly varied from 0 to −8 m/year. The other two intercepts, β_2_ and β_3_, were solved for such that the melt function is piecewise continuous. The smoothing function uses a randomly selected smoothing length, γ, which ranges from 200 to 4000 m. There are two smoothing modes, which are equally likely to be applied. The first continues the melt function, with thickness data inland of the grounding line, and smooths the data across the grounding line, which tends to maximize melt at the grounding line. The second mode imposes zero melt at the grounding line, which forces the maximum melt to be higher in the water column (see examples of each in Fig. 1). Last, a scaling factor, ξ, is updated at each step to match the prescribed melt, *M*(*t*), which, in our simulations, could be a constant (e.g., 57, 75, 100, and 125Gt/year), sinusoidally varying about a mean or a linear function of time.

### Shelf reduction experiment

The simulations are referenced to 2017 before a series of calving events reduced the length of the ice shelf by nearly 20 km (*25*). As the shelf front retreated, the near grounding line speeds that had been relatively stable for nearly a decade began to increase.

To simulate the effect of the recent calving, we produced a new mesh that represents the nominal 2020 shelf-front position (fig. S4). We then regridded the 2017 initial solution to this mesh. As a result, the simulation starts up as though there was an abrupt calving event that removed nearly 20 km of the shelf (20% reduction in area), rather than the more phased retreat that actually occurred over a 3-year period. Three years after the shelf removal, the simulated speeds near the grounding line agree well with the late 2020 speeds for both the 57 and 75 Gt/year melt scenarios (*25*), suggesting that melt rates in this range are consistent with current conditions.

### Comparison with ISMIP6 results

ISMIP6 performed numerous simulations for Antarctica for which they broke out the losses by drainage basin for the years 2015 to 2100 as a function of sub–ice-shelf melt (*13*) for all of the Representative Concentration Pathway 8.5 experiments with medium ocean conditions. We extracted these results from the ISMIP6 repository (*49*) for the five basins with the largest losses (mean loss across all simulations, >3 mm sle), which are shown in Fig. 5. This cutoff was chosen to avoid cases where any signal due to melt would likely be below the intermodel spread. The ISMIP6 results are all expressed relative to their respective control runs. Thus, Fig. 5 shows ice loss and melt anomalies relative to the corresponding values from the control runs.

For comparison, we performed a similar regression for our 85-year losses and found a similar sensitivity (black line in Fig. 5). Although we did not have a formal control run, we plotted our results relative to the 57 Gt/year result to facilitate comparison. We note that due to the linearity, this sensitivity should be the same or similar for a population of identical or similar glaciers as for an individual glacier (e.g., two PIG-like glaciers with half the melt should produce the same loss as a single PIG with full melt).
