# Supplementary material for: Ocean-induced melt volume directly paces ice loss from Pine Island Glacier
Source: Sci Adv. 2021 Oct 22;7(43):eabi5738. doi: 10.1126/sciadv.abi5738 (PMC8535793; doi:10.1126/sciadv.abi5738)
Supplement: Supplementary file 1 — Figs. S1 to S4 [file sciadv.abi5738_sm.pdf]

Science Advances
AAAS

Supplementary Materials for

Ocean-induced melt volume directly paces ice loss from Pine Island Glacier

Ian Joughin*, Daniel Shapero, Pierre Dutrieux, Ben Smith

*Corresponding author. Email: ian@apl.washington.edu

Published 22 October 2021, *Sci. Adv.* **7**, eabi5738 (2021)
DOI: 10.1126/sciadv.abi5738

This PDF file includes:

Figs. S1 to S4

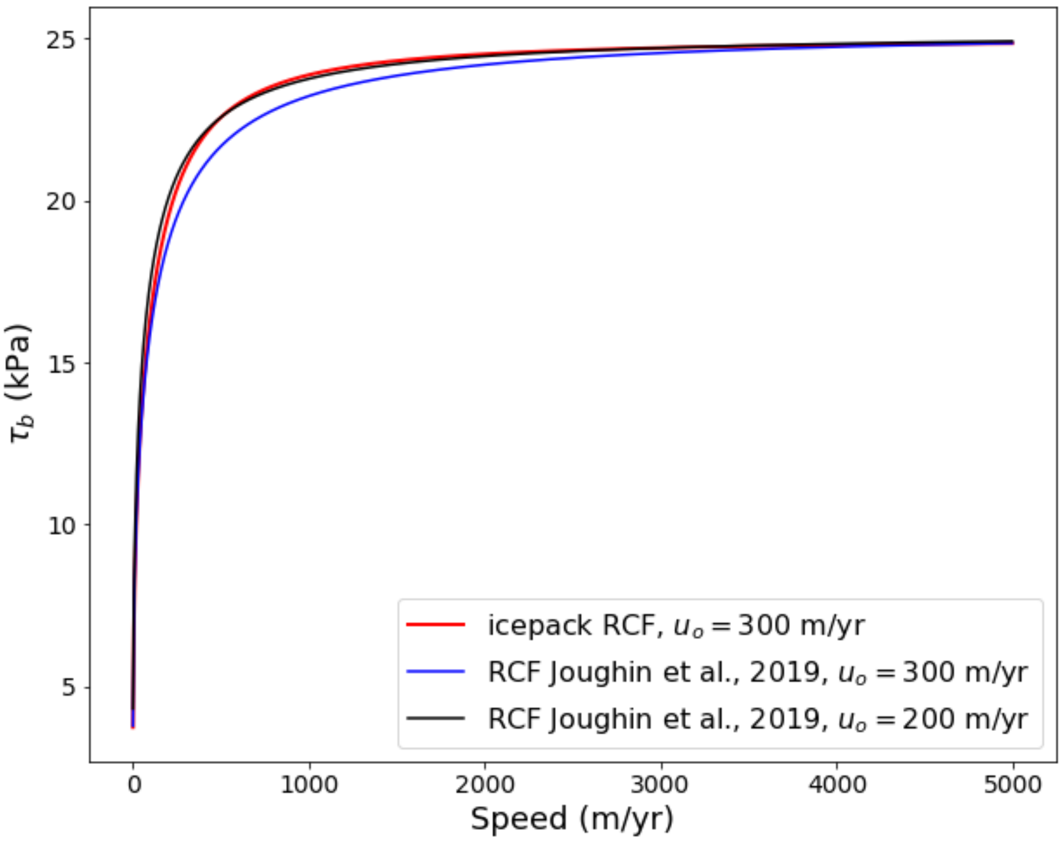

Fig. S1.

Functions shown in Equations 1 (blue) and 2 (red) with C adjusted to produce the same arbitrarily selected values at high speeds with $u_o = 300$ m/yr. Also shown is a second result ($u_o = 200$ m/yr) computed using Equation 1, which agrees more closely with the result from Equation 2.

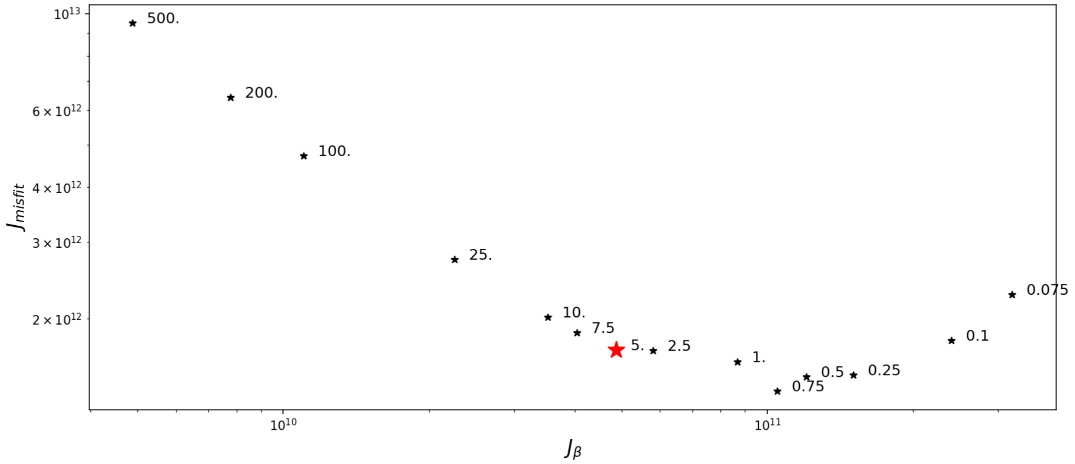

Fig. S2

L-Curve showing model-data misfit, J_{misfit}, as a function of the regularization term, J_β, as determined by the Tikhonov regularization coefficient, $\alpha 2.1125E10$. The numerical scale factor was selected to produce reasonable values when α is near unity. The final inversions were performed using $\alpha = 5$ (red star).

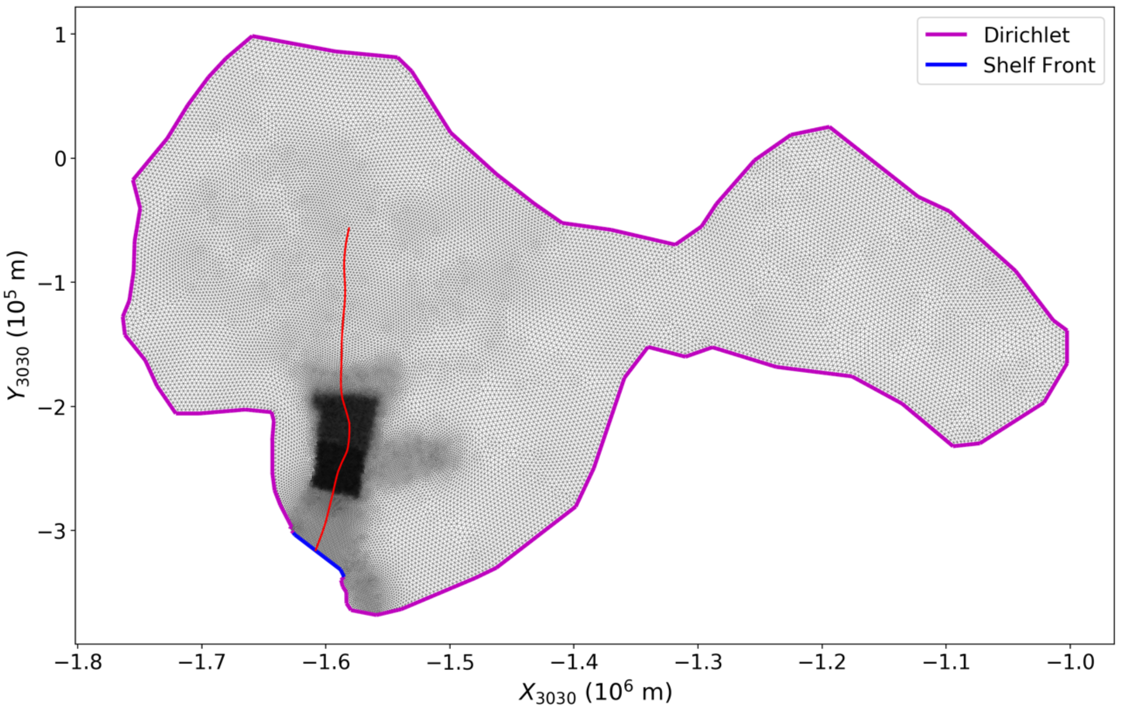

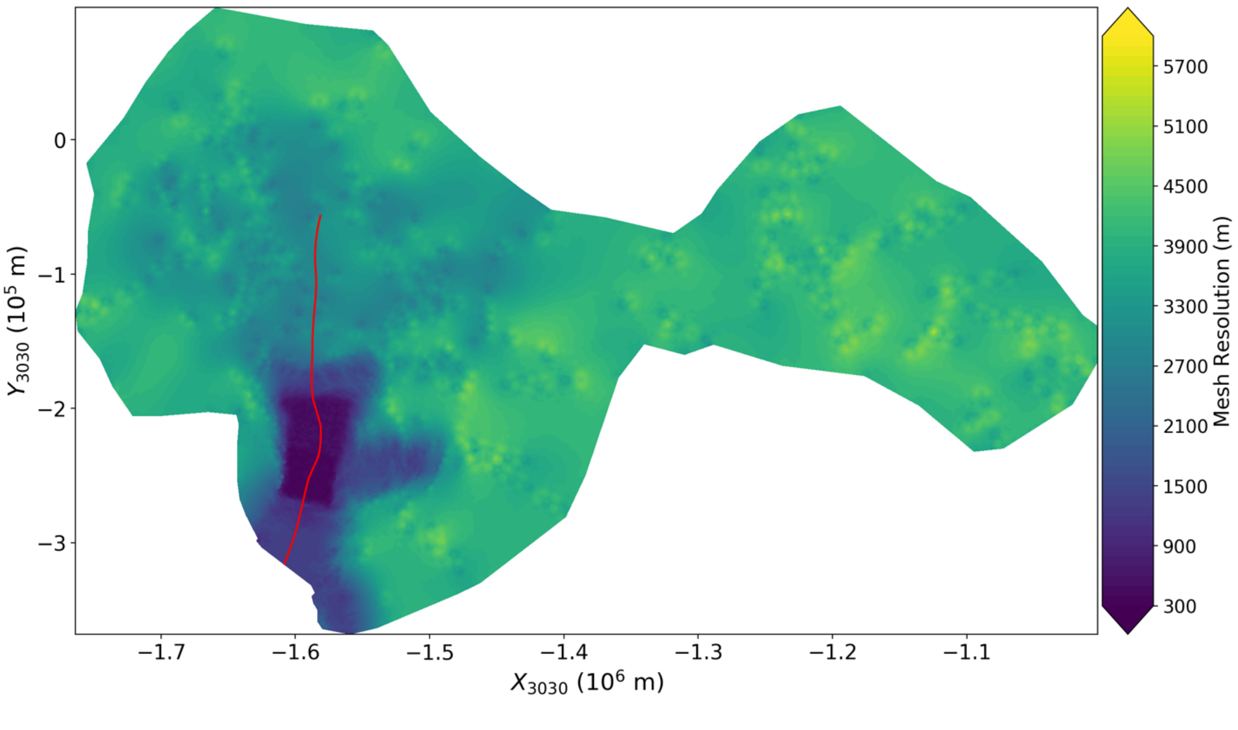

Fig. S3.

Pine Island Glacier (top) finite element mesh and (bottom) mesh resolution.

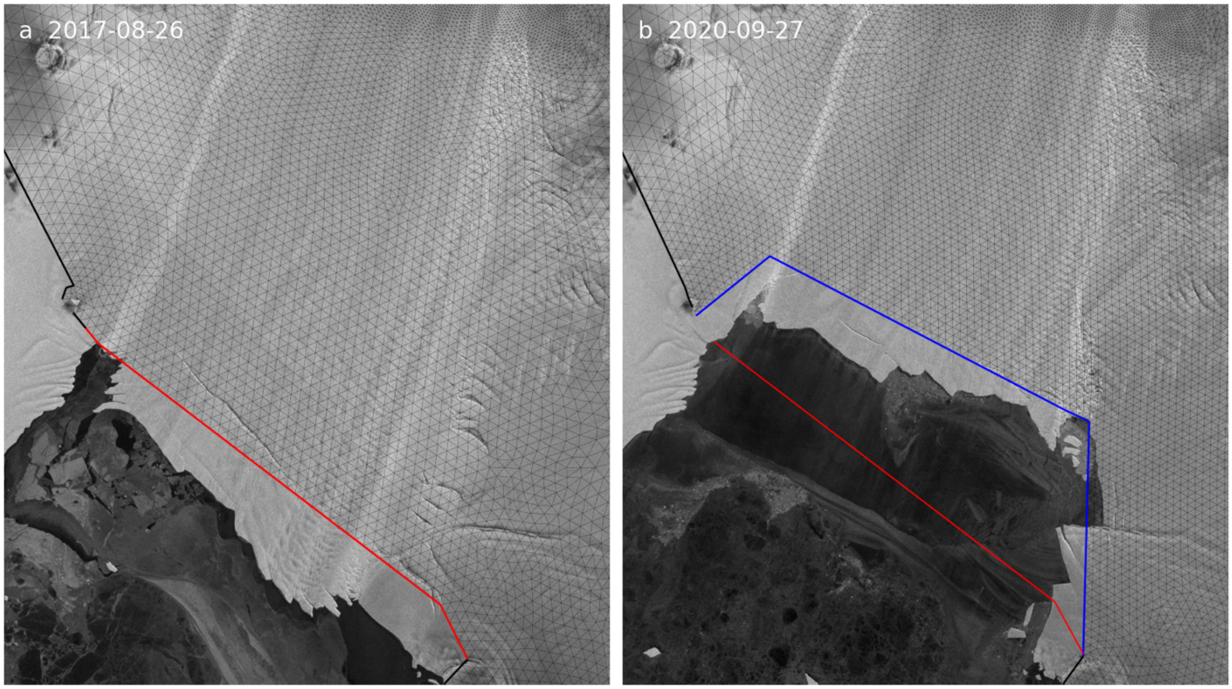

Fig. S4.

Portion of the model meshes covering the outer PIG ice shelf for the a) 2017 and b) 2020 cases plotted over Sentinel 1A/B images from the corresponding years. Red and blue lines show the simulated calving fronts for 2017 and 2020, respectively.
